# RSF-1 overexpression determines cancer progression and drug resistance in cervical cancer

**DOI:** 10.1051/bmdcn/2018080104

**Published:** 2018-02-26

**Authors:** Xiangyu Wang, Jim Jinn-Chyuan Sheu, Ming-Tsung Lai, Cherry Yin-Yi Chang, Xiugui Sheng, Ling Wei, Yongsheng Gao, Xingwu Wang, Naifu Liu, Wenli Xie, Chih-Mei Chen, Wendy Y. Ding, Li Sun

**Affiliations:** 1 School of Medicine and Life Sciences, University of Jinan-Shandong Academy of Medical Sciences Jinan 250022 China; 2 Department of Gynecological Oncology, Shandong Cancer Hospital Affiliated to Shandong University, Shandong Academy of Medical Sciences Jinan 250117 China; 3 Institute of Biomedical Sciences, National Sun Yat-sen University Kaohsiung 804 Taiwan; 4 Human Genetic Center, China Medical University Hospital Taichung 404 Taiwan; 5 School of Chinese Medicine, China Medical University Taichung 404 Taiwan; 6 Department of Pathology, Taichung Hospital, Ministry of Health and Welfare Taichung 403 Taiwan; 7 Maternity department, China Medical University Hospital Taichung 404 Taiwan

**Keywords:** RSF-1 (HBXAP), Cervical cancer, Clinical pathological, characteristics, Anti-RSF-1 therapy

## Abstract

Background: Remodeling spacing factor 1 (RSF-1/HBXAP) has been linked to a variety of cancer types, however, its roles and the therapeutic potential are not clear in cervical cancer.

Methods: RSF-1 expression in cancer tissues was analyzed by immunohistochemical staining followed by statistical analysis with SPSS. Anti-RSF-1 studies were performed by treating cells with specific siRNA or a dominant mutant form (RSF-D4).

Results: RSF-1 expression correlates with cancer progression that strongly-positive staining can be found in 67.7% carcinomas and 66.7% CIN lesions, but none in normal tissues. Such overexpression also associated with increased tumor size, poor differentiation, higher nodal metastasis and advanced clinical stages. Kaplan– Meier analysis confirmed that cancer patients with high RSF-1 levels exhibited a significantly shorter survival time than those with low RSF-1 levels. Downregulation of RSF-1 by siRNA silencing or RSF-D4 reduced cell growth and increased drug sensitivity toward paclitaxel treatment in HeLa cells.

Conclusions: RSF-1 participates in the tumor progression of cervical cancer and could be considered as an early prognostic marker for cancer development and clinical outcome. Therapies based on anti-RSF-1 activity may be beneficial for patients with RSF-1 overexpression in their tumors.

## 1. Introduction

Cervical cancer is one of the most common gynecological malignancies and the fourth prevalent cause of cancer-related death in women [1]. It is a heterogeneous multifocal disease and the incidence rate is increasing. The mechanisms that influence the oncogenesis and progression of cervical cancer include multi-step processes, which involve both gene tampering in cervical epithelial cells and infection of HPV [2]. Although several relatively effective therapies, such as platinum-based chemo drugs and concurrent chemoradiotherapy (CCRT), are available for treating patients, locoregional recurrence-free survival (LRFS), diseasefree survival (DFS) and overall survival (OS) are still low and need to be improved. Due to such limitations, it is necessary to identify other molecular effectors that influence oncogenesis of cervical cancer in order to find more effective and safe strategies or therapeutic avenues for preventing and curing cervical cancer.

It has been known that genomic DNA existing in eukaryotic nuclei is closely packaged and organized with histone proteins into the basic frame of chromatin, nucleosomes. Chromatin remodeling factors are found to participate in regulation of chromatin structure dynamics through DNA packaging and chromatin winding/unwinding, providing the access for other nuclear proteins [3], which subsequently controls DNA synthesis, gene transcription and DNA damage repair. Thus, chromatin remodeling factors play important roles in many cellular processes, which determine tissue development and differentiation, as well as the pathogenesis of various diseases including cancer [4, 5]. Based on previous findings, there are at least three main families related to chromatin remodeling complexes, including the ISWI, SWI/SNF, and CHD/Mi-2 families [6]. These chromatin remodelers possess differential binding affinities toward distinct specific nucleosome positions and establish unique chromatin structures [7]. Genetic alterations or mutations in chromatin remodeling factors have been frequently identified in various cancers by the cancer genome project and recognized as one of main driving forces for cancer development [8, 9].

RSF-1 (also called HBXAP) was previously found as the key cancer-driving gene within the defined 11q13.5 genetic amplicon in ovarian cancers [10], which can be supported by several other studies in other cancer types. Results from these studies came to a similar conclusion that the expression levels of RSF-1 in human tissues correlated with advanced cancer progression and can serve as a prognostic marker for poor clinical outcomes and shorter survival rate [11-18]. RSF-1 overexpression was also found to contribute to paclitaxel resistance [19], and recurrent tumors after chemo- or radiotherapy showed higher Rsf-1 levels [17]. By contrast, RSF-1 knockdown by siRNA treatment or functional competition with deletion mutants reduced cell growth, increased drug sensitivity and triggered cell death in cancer cells with RSF-1-overexpression [19, 20]. In addition, the RSF complex (RSF-1 with SNF2H) can collaborate with cyclin E, another well-known cancer-driver, to trigger more aggressive cancer behaviors through promoting G1-S transition [21]. Recent studies further identified the involvement of RSF-1 in DNA recombination and unequal chromosome segregation that may result in genome instability [22-24].

With solid evidence showing the involvement of RSF-1 in cancer development, however, there are few reports regarding its roles in cervical cancer. Our study therefore aims to identify potential effects of RSF-1 overexpression in cervical cancer by immunohistochemistry. The association between RSF-1 levels and clinical pathological characteristics were analyzed to know its prognostic potential. Finally, human cervical cancer HeLa cells were used as an experimental model to verify the efficacy of anti- RSF-1 therapy for treating cervical cancer.

Materials and Methods

### 2.1. Patients and tissue collection

A total of 160 cervical cancer tissues were obtained from Shandong Cancer Hospital Affiliated Shandong University between 2000 and 2016. In addition, 40 cases of cervical intraepithelial neoplasia (CIN) and 20 normal cases were enrolled. The clinical stage and histological diagnosis were identified on the basis of the International Federation of Gynecology and Obstetrics (FIGO) classification system. Follow-up information was collected every three months *via* telephone, during reexamination or by mail. Acquisition of tissue specimens and clinical information were approved by Ethics Committee of the hospital with signed consents from patients.

### 2.2 Immunohistochemical staining and scoring

Antigen retrieval of tissue sections was performed using a standard protocol and the resultant sections were incubated with anti RSF-1 rabbit monoclonal antibody (1:250 dilution; Abcam, USA) followed by goat anti-rabbit serum IgG. (1:250 dilution; Abcam, USA). Two investigators examined all tumor slides randomly. Nuclear immunostaining in tumor cells was considered as positive staining. Based on previous reports [12, 13], the RSF-1 in each tissue was scored from 0 to 12 *via* multiplying the staining extent and intensity of nuclear staining. Subsequently, the tumor samples with a score of 6+-12+ were determined as RSF-1 high expression and the samples with a score of 9+-12+ were determined as RSF-1 low-expression. To reduce possible bias, tissue slides with inadequate staining were excluded.

### 2.3. Cell lines and cell culture

HeLa, SiHa, and 293T cells were obtained from Shandong Cancer Hospital Affiliated Shandong University and cultured in RPMI-1640 containing 10% fetal calf serum, 100 IU/*ml* penicillin, and 100 μg/*ml* streptomycin. For growth assay, cells were grown at a density of 3,000 cells per well in 96-well plates. After overnight culture, the cells were treated with paclitaxel, specific anti-RSF-1 siRNA [10] or RSF-1 dominant negative form (RSF-D4) [19, 20], and cell growth was monitored daily for 4 consecutive days based on fluorescence intensity of SYBR Green I nucleic acid staining (Molecular Probes, Eugene, OR).

### 2.4. Quantitative real-time PCR

Total RNA was extracted from cells using Trizol. Quantitative real-time PCR was done using SYBR Green PCR Master Mix (Kang century Biotechnology Co., Beijing, China). The sequences of the primer pairs are as follows: RSF-1 forward, 5’ GATACTATGCGTCTCCAGCCAA 3’, RSF-1 reverse, 5’, CAACTCGTTTCGATTTCTGACAA 3’; β-actin forward, 5’ ATAGCACAGCCTGGATAGCAACGTAC 3’, β-actin reverse, 5’ CACCTTCTACAATGAGCTGCGTGTG 3’. β-actin was used as the reference gene.

### 2.5. Western blot analysis

Total proteins from cells were extracted in lysis buffer (Kang century Biotechnology Co.) and quantified using the Bradford method (Kang century Biotechnology Co.). Samples of 60 μg protein were separated by SDS-PAGE and transferred to Polyvinylidene fluoride membranes. After blocking, the membranes were incubated overnight at 4°C with antibodies against RSF-1 (1:1000, Kang century Biotechnology Co.) or rabbit monoclonal antibodies against β-actin (1:1000, Kang century Biotechnology Co., Ltd.). After incubation with peroxidase-coupled anti-rabbit IgG (1:1000, Kang century Biotechnology Co.) at 37°C for 2h, bound proteins were visualized using ECL substrate (1:1000, Kang century Biotechnology Co.) and detected using a BioImaging System (1:1000, Kang century Biotechnology Co.).

### 2.6. Statistical analysis

SPSS version 21.0 for Windows (IBM) was used for all statistical analyses. The χ2 test and Fisher test were used to examine correlations between RSF-1 expression and the clinical characters. Cox’s proportional hazards model was used to identify the statistic significance. All *p*-values were based on the two-sided statistical analysis and *p* < 0.05 was considered to be statistically significant.

## 3. Results

### 3.1. Association study of RSF-1 immunohistochemical staining with clinical pathological characteristics

Patient demographics and tumor characteristics in this study are summarized in Table 1. Tumor histology analysis indicated that 30.0 % of total cancer patients were diagnosed as adenocarcinomas and 70.0 % of them were squamous carcinomas. Cancer patients sub-grouped by tumor stage were as follows: FIGO stage I, 6.25 %; stage II, 26.25 %; stage III, 34.38 %; and stage III, 33.12 %, for all patients who had surgery as the first clinical intervention.

**Table 1:** Summary of clinical characteristics of the study cohort.

Variable	Total no. of patients	
	**(n)**	**Proportion (%)**
**Age (mean ± SD)**	51±10.6	
≤ 32	9	4.09%
33-50	91	41.36%
≥ 51	120	54.55%
**Normal tissues**	20	
**Cervical intraepithelial neoplasia (CIN)**	40	
CIN I	5	12.50%
CIN II	14	35.00%
CIN III	21	52.50%
**Cervical cancer**	160	
Tumor size(cm)	160	
≤4	90	56.25%
>4	70	43.75%
FIGO stage	160	
I	10	6.25%
II	42	26.25%
III	55	34.38%
IV	53	33.12%
Histological grade	160	
Well differentiated	43	26.88%
Moderately differentiated	44	27.50%
Poorly differentiated	73	45.62%

Statistical significance. (*: *P* < 0.05; **: *P* < 0.01; ***: *P* < 0.001)

**Fig. 1:**
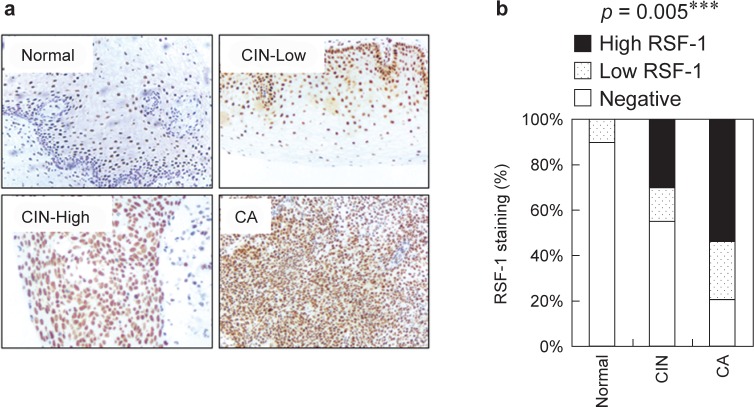
RSF-1 expression in different cervical tissues. (a) RSF-1 staining was performed and scored on different cervical tissues. Representative staining images for normal tissues, CINs and cervical carcinomas were presented. (b) RSF-1 strongly-positive staining is more frequent in carcinomas and CINs than in normal.

**Fig. 2:**
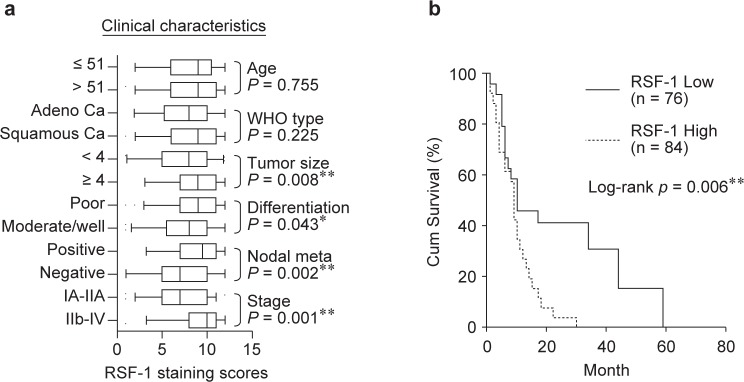
RSF-1 expression correlates with poor clinical outcomes. (a) Samples with high RSF-1 expression (staining scores of 9+ to 12+) tends to be associated with bigger tumor sizes, poor differentiation, nodal metastasis and more advanced stages. (b) Kaplan-Meier survival analysis shows a shorter overall survival for patients with high RSF-1 expression.

To gain insight into the effects and prognostic values of RSF-1 expression, 220 tissue sections, including 160 tumors, 40 CINs and 20 normal tissues, were examined by immunohistochemistry. Seventy-three cases, 18 of normal tissues (90.0 %), 22 CINs (55.0 %) and 33 (20.6 %) carcinomas, were found to show undetectable RSF-1 expression (Fig. 1A). Other 147 cases showed a wide range of positive staining in tumor nuclei in which 98 cases were scored as 9+ to 12+ and 49 cases were scored as 6+ to 8+, respectively (Fig. 1A). As compared to normal and CIN tissues, RSF-1 protein was significantly upregulated in malignant cervical carcinomas, as shown in Fig. 1B (*p* = 0.0005). In particular, RSF-1 overexpression (scores of 9+ to 12+).correlates with bigger tumor size (*p* = 0.0015), poor differentiation (*p* = 0.0097), nodal metastasis (*p* = 0.0001), and tumor stages (*p* < 0.0001) (Table 2 and Fig. 2A).

**Table 2:** Distribution of RSF-1 status in human cervical cancer according to clinicopathological characteristics.

Characteristics	Number	RSF-1 over-expression (n = 86)	Negative/weak RSF-1 expression (n = 76)	P values
**Age (years)**				**0.9285**
≤51	74	39	35	
>51	88	47	41	
**Tumor size (cm)**				0.0016**
<4	81	33	48	
≥4	81	53	28	
**Tumor Differentiation**				0.0098**
poorly differentiated	75	48	27	
moderately and well differentiated	87	38	49	
**Nodal metastasis**				0.0002***
Positive	83	56	27	
Negative	79	30	49	
**Tumor Stage**				<0.0001***
IA-IIA	90	35	55	
IIB-IV	72	52	21	

Statistical significance (*: P < 0.05; **: P < 0.01; ***: P < 0.001)

### 3.2. RSF-1 expression level correlates with poor clinical outcome

Previous studies on a variety of tumor types have shown the correlation between RSF-1 overexpression and poor clinical outcomes in cancer patients. We, therefore, stratified cases into two groups: the first group with low RSF-1 expression (scores of 0 to 8+), and the other with high RSF-1 expression (scores of 9+ to 12+). Consistent with previous studies, patients with high expression levels of RSF-1 had a shorter overall survival time (30 months) than did patients with low RSF-1 levels (59 months) (Fig. 2B). Our data suggest RSF-1 overexpression a prognosis marker for cervical cancer patients with poor clinical outcomes.

### 3.3. RSF-1 overexpression in cervical cancer cell lines and biological effects of anti-RSF-1 treatments on cell growth

**Fig. 3:**
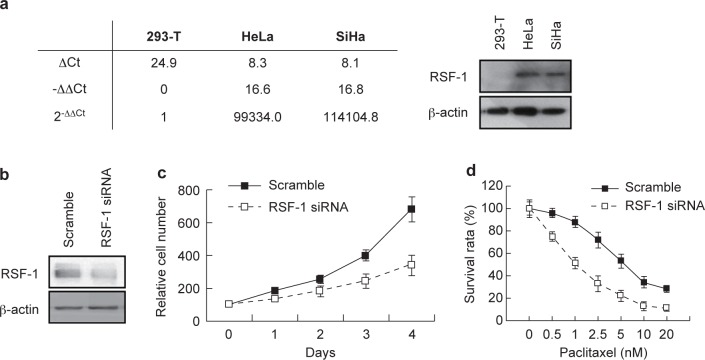
Impacts of RSF-1 knockdown by specific siRNA. (a) RSF-1 levels in HeLa and SiHa cells were detected by QPCR (left) and Western blotting (right) using 293-T cells as the control. (b) RSF-1knockdown efficiency was verified by Western blotting. RSF-1 knockdown (c) reduced cell growth and (d) increased paclitaxel sensitivity in HeLa cells.

**Fig. 4:**
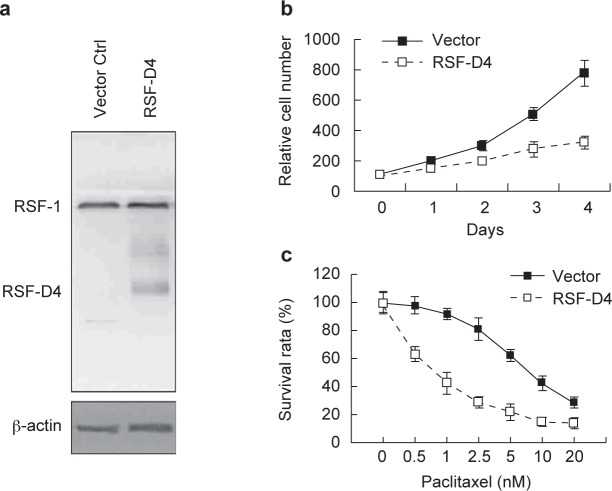
Functional impacts of the dominant negative RSF-1 (RSF-D4). (a) Western blotting was performed to detect RSF-D4 expression in HeLa cells. Cells transfected with empty vectors were used as the negative control. RSF-1 functional knockdown by RSF-D4 (b) reduced cell growth and (c) increased paclitaxel sensitivity in HeLa cells.

To assess the possibility of using RSF-1 as a therapeutic target, we detected its mRNA expression levels in cervical cancer cell lines (HeLa and SiHa) using 293T cells as the control, a well known non-tumorigenic cell line. Quantitative real-time PCR confirmed more than 10,000 folds of mRNA levels of RSF-1 in HeLa and SiHa cells as compared with the level in 293T cells (Fig. 3A, *left*). Western blot also demonstrated robust RSF-1 expression in these two cervical cancer cell lines (Fig. 3A, *right*). We next used HeLa cells as the model cell line to investigate the biological impacts of RSF-1 downregulation. Firstly, we knocked down RSF-1 expression by specific siRNA (Fig. 3B) and found that the cell growth rate was significantly reduced (Fig. 3C). Secondly, we introduced an RSF-1 dominant negative form RSF-D4 to inhibit the formation of functional RSF complex [19, 20] (Fig. 4A) and also found suppressive effects on cell growth (Fig. 4B). To know whether RSF-1 overexpression determines drug sensitivity in cervical cancer cells, we estimated the IC_50_ of paclitaxel treatment in HeLa cells with or without RSF-1 down regulation. As shown in Figures 3D and 4C, cells treated with either specific siRNA or RSF-D4 became more sensitive toward paclitaxel treatment with lower IC50. Our results thus supported the view that RSF-1 overexpression functions as a driving force to promote cell growth and control drug sensitivity toward paclitaxel treatment.

## 4. Discussion

In this study, we evaluated RSF-1 expression by immunohistochemical assay. RSF-1 expression was significantly increased in cervical cancer tissues than in CIN and normal tissues. We also examined whether RSF-1 overexpression is a predictive marker of more aggressive behaviors by correlating IHC score to clinicopathologic variables. In agreement with previous studies on other gynecological cancers, such as ovarian high-grade serous carcinoma [10, 15] and ovarian clear cell carcinoma [18], up-regulation of RSF-1 is related to bigger tumor sizes, poor cytopathological characteristics, advanced stages and nodal metastasis. In addition, overexpression of RSF-1 in cervical cancer predicts a shorter survival time, suggesting its prognostic value for clinical application.

Paclitaxel, an inhibitor of microtubule polymerization, is one of the most useful anticancer drugs for treating cervical cancer at clinics. Although paclitaxel-based therapy shows impressive initial clinical responses, the major failure for treating patients is eventually due to the development of some degree of drug resistance. In this study, we discovered that RSF-1 overexpression provided survival strength for HeLa cells to conquer paclitaxel effects, thus functional knockdown by specific siRNA can render the cells more sensitive toward drug treatment. Consistent with the previous study [19, 20], our findings with the dominant negative form RSF-D4 also revealed essentials of a functional RSF complex comprised of both RSF-1 and SNF2H for drug resistance phenotype. Our data indicate RSF-1 overexpression not only a useful biomarker for clinical prognosis but also a potential target for patient treatment.

Increased RSF-1 expression was also reported in other solid tumors including gastric adenocarcinoma [16] and oral cancer [17]. Therefore, the molecular programs driven by RSF-1 overexpression are interesting topics to explore. Multiple studies have demonstrated the tumor-promoting interplays between RSF-1 and known oncogenes or tumor suppressors, such as cyclin D1 [11], cyclin E1 [21], retinoblastoma (RB) [12, 25] and breast cancer susceptibility gene 1 (BRCA1) [24]. Since RSF-1 possesses a PHD domain, a sequence motif for Zinc finger, these results suggest a potential role for RSF-1 to regulate gene transcription in cervical epithelium during carcinogenesis. In particular, RSF-1 was originally discovered as a viral protein binding protein [26, 27], whether RSF-1 can form interactive networks with HPVencoded oncoproteins remains further investigation.

In conclusion, our data suggest that chromatin remodeling factor, RSF-1, participates in the tumor progression of cervical cancer and could be considered as a prognostic marker for predicting clinical outcome. Targeting RSF-1 gene expression and the pathway it controls may provide new therapeutic avenues for treating advanced stage cervical cancer that are refractory to conventional therapy.

## Compliance with ethical standards

All procedures performed in studies involving human participants were in accordance with the ethical standards of the institutional and/or national research committee (SDTHEC) and with the 1964 Helsinki declaration and its later amendments or comparable ethical standards.

## Conflict of interest statement

The authors declared no conflict of interest in this study.
